# Analysis of Metals Concentration in the Soils of SIPCOT Industrial Complex, Cuddalore, Tamil Nadu

**DOI:** 10.4103/0971-6580.72681

**Published:** 2010

**Authors:** V. Mathivanan, R. Prabavathi, C. Prithabai, Selvisabhanayakam

**Affiliations:** Department of Zoology, Annamalai University, Annamalai Nagar - 608 002, Tamil Nadu, India

**Keywords:** Metal concentration, SIPCOT, soil, Tamil Nadu

## Abstract

Phytoremediation is a promising area of new research, both for its low cost and great benefit to society in the clean retrieval of contaminated sites. Phytoremediation is the use of living green plants for *in situ* risk reduction and/or removal of contaminants from contaminated soil, water, sediments, and air. Specially selected or engineered plants are used in the process. The soil samples were taken from Cuddalore Old Town (OT) and the samples from SIPCOT industrial complex, which was the study area and analyzed for various metals concentrations. Fifteen metals have been analyzed by adopting standard procedure. The detection limits of metal concentration are drawn as control. The various (15) metal concentrations in the soil samples were found higher in soil taken from SIPCOT industrial complex, compared with samples taken from Cuddalore OT. In all the observations, it was found that most of the metals like calcium, cadmium, chromium, cobalt, nickel, and zinc showed maximum concentrations, whereas arsenic, antimony, lead, magnesium, sodium have shown minimum concentrations, both when compared with control. From the present study, it was found that the soil collected from SIPCOT complex area were more polluted due to the presence of various industrial effluents, municipal wastes, and sewages when compared with the soil collected from Cuddalore OT.

## INTRODUCTION

Over centuries, human industrial, mining, and military activities as well as farm practices have contaminated large areas of developed countries with high concentration metals of organic pollutants. In addition to their negative effects on ecosystem resources, these sites pose a great danger to public health, because pollutants and agricultural products are leached into drinking water.[1,2]

In India, for instance, only contaminated sites are cleaned up in soil remediation facilities;[3] that be stored in waste disposal facilities. This does not solve the problem, it merely generates problems. Obviously, there is an urgent need for alternative, cheap, and efficient methods to clean up heavily contaminated industrial areas.

This could be achieved by a relatively new technology known as phytoremediation by plants to remove pollutants from the environment. Due to its presence technology, this metal has contaminated all the areas, it has already received significant scientific and commercial attention.[4–10] Most scientific and commercial interest in phytoremediation now focuses on phytodegradation, which use selected plant species grown on contaminated soil harvested to remove the plants together with the pollutants that have accumulated. Depending on the nature of contamination, the plants can either be disposed off or processed by burning for energy production. In essence, phytoextraction from contaminated soils concentrates them in biomass and further concentrates to be removed by combustion.

Plants, with some having the ability to uptake heavy metals, could be employed to clean up old mining sites and other sites contaminated with heavy metals. The experimental plants have been harvested, dried, and burned. The organic material would burn off leaving deposits of heavy metals that could be recycled.

Phytoremediation is a promising area of new research, both for its low cost and great benefit to society in the clean retrieval of contaminated sites. The first goal in phytoremediation is to find a plant species which is resistant to/or tolerates a particular contaminant with a view to maximizing its potential for phytoremediation. Resistant plants are usually located growing on soils with underlying metal ores or on the boundary of polluted sites. Once a tolerant plant species has been selected, traditional breeding methods are used to optimize the tolerance of a species to a particular contaminant.

### Application of phytoremediation

All of these technologies involve relatively high capital expenditure and manpower as well as long-term operating costs. Therefore, efforts are underway to develop more cost-effective approaches to treat large volumes of contaminated natural resources such as soil, ground water, and wetlands.[11,12] Currently, phytoremediation is used for treating many classes of contaminants including petroleum hydrocarbons, chlorinated solvents, pesticides, explosives, heavy metals and radionuclides, and landfill leachates. In India, terrestrial plants like *Helianthus annuus, Phragmites karka, Datura innoxia, Brassica juncea, Alternanthera sessilis*, and *Zea mays* have been used to treat different metals contaminating effluent soil and sludge from various types of industries.[6,8,10,12] In view of the foregoing literature, it is programmed to make an attempt to study the nature of phytoremediation technique in the soils of study area by using the terrestrial plant, *Helianthus annuus*.

## MATERIALS AND METHODS

The studies were carried out over a period of three months, that is, between January 2008 and March 2008. Samplings of soil from study area were made for 20 days and 40 days only. The method used for plant digestion in the present study was followed as described by Al-Shayeb *et al* 1995.[13] The soil samples were taken from Cuddalore Old Town (OT) and the samples from SIPCOT industrial complex, which were the study area. The soil samples were analyzed for various metal concentration.

## RESULTS AND DISCUSSION

**Figures 1-15:**
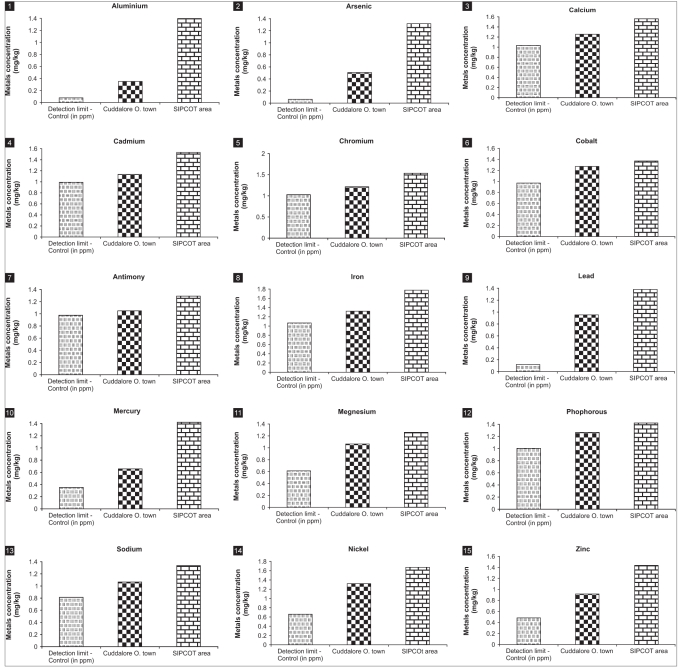
Metal concentration (mg/kg) in soils of Cuddalore OT and SIPCOT industrial complex area

A total of 15 metals have been analyzed by adopting standard procedure. The detection limits of metal concentration are drawn as control. The various (15) metal concentrations in the soil samples were found higher in soil taken from SIPCOT industrial complex, compared with samples taken from Cuddalore OT. In all these observations, it was found that most of the metals like calcium, cadmium, chromium, cobalt, nickel, and zinc showed maximum concentrations, whereas arsenic, antimony, lead, magnesium, sodium have shown minimum concentrations, both when compared with control. All the observations are shown in Table 1 and Figures 1 to 15.

**Table 1:** Trace metal concentration in the soils (mg/kg of dry soil) of Cuddalore OT and SIPCOT complex area, Tamil Nadu

Metals	Detection limit -Control	Cuddalore OT	SIPCOT Area
Aluminium (Al)	0.08	0.35	1.40
Arsenic (As)	0.06	0.50	1.32
Calcium (Ca)	1.03	1.25	1.55
Cadmium (Cd)	1.04	1.18	1.60
Chromium (Cr)	1.02	1.20	1.85
Cobalt (Co)	1.03	1.35	1.45
Antimony (Sb)	0.98	1.05	1.30
Iron (Fe)	1.05	1.30	1.75
Lead (Pb)	0.12	0.95	1.38
Mercury (metal)	0.35	0.65	1.40
Magnesium (Mg)	0.61	1.05	1.25
Phosphorus (P)	1.00	1.25	1.40
Sodium (Sa)	0.80	1.05	1.32
Nickel (Ni)	0.65	1.30	1.65
Zinc (Zn)	0.50	0.95	1.50

OT – Old Town

In contrast to many organic pollutants which are anthropogenic and often degraded in the soil, metals occur naturally and are conserved.[14] Due to their immutable nature, heavy metals are a group of pollutants of much concern. The danger of heavy metals is aggravated by their almost indefinite persistence in the environment.[3,6,9] Although some metals are essential for life (i.e., they provide essential cofactors for metalloproteins and enzymes), at high concentrations they can act in deleterious manner by blocking essential functional groups, displacing other metal ions, or modifying the active conformation of biological molecules.[2,15]

As a consequence of the alteration of its oxidation state, the metal may become either: (i) more water soluble and is removed by leaching, (ii) inherently less toxic, (iii) less water soluble so that it precipitates and then becomes less bioavailable or removed from the contaminated site, or (iv) volatilized and removed from the polluted area. Heavy metals are present in soil as natural components or as a result of human activity. The primary sources of metal pollution are the burning of fossil fuels, mining, and smelting of metalliferous ores, down wash from power lines, municipal wastes, fertilizers, pesticides, and sewage.

From the present study, it was found that the soil collected from SIPCOT complex area was more polluted due to the presence of various industrial effluents, municipal wastes and sewages, drainage, when compared with the soil collected from Cuddalore OT.
